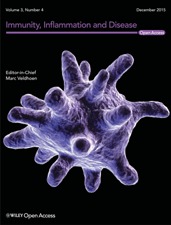# Issue Information

**DOI:** 10.1002/iid3.31

**Published:** 2015-11-26

## Abstract